# Design and Optimization of FBG Implantable Flexible Morphological Sensor to Realize the Intellisense for Displacement

**DOI:** 10.3390/s18072342

**Published:** 2018-07-19

**Authors:** Changbin Tian, Zhengfang Wang, Qingmei Sui, Jing Wang, Yanan Dong, Yijia Li, Mingjuan Han, Lei Jia, Hanpeng Wang

**Affiliations:** 1 School of Control Science and Engineering, Shandong University, Jinan 250061, China changbin_tian@163.com (C.T.); qmsui@sdu.edu.cn (Q.S.); dongyanan0115@163.com (Y.D.); liyijiasdu@163.com (Y.L.); han1772824165@126.com (M.H.); jialei@sdu.edu.cn (L.J.); 2 Geo & Stru Engineering Research Center, Shandong University, Jinan 250061, China; whp@sdu.edu.cn

**Keywords:** FBG flexible sensor, morphological sensing, classification morphological correction method, conjugate gradient method, ELM

## Abstract

The measurement accuracy of the intelligent flexible morphological sensor based on fiber Bragg grating (FBG) structure was limited in the application of geotechnical engineering and other fields. In order to improve the precision of intellisense for displacement, an FBG implantable flexible morphological sensor was designed in this study, and the classification morphological correction method based on conjugate gradient method and extreme learning machine (ELM) algorithm was proposed. This study utilized finite element simulations and experiments, in order to analyze the feasibility of the proposed method. Then, following the corrections, the results indicated that the maximum relative error percentages of the displacements at measuring points in different bending shapes were determined to be 6.39% (Type 1), 7.04% (Type 2), and 7.02% (Type 3), respectively. Therefore, it was confirmed that the proposed correction method was feasible, and could effectively improve the abilities of sensors for displacement intellisense. In this paper, the designed intelligent sensor was characterized by temperature self-compensation, bending shape self-classification, and displacement error self-correction, which could be used for real-time monitoring of deformation field in rock, subgrade, bridge, and other geotechnical engineering, presenting the vital significance and application promotion value.

## 1. Introduction

Fiber Bragg grating (FBG) is a promising sensing element for the fabrication of flexible sensors owing to its advantageous characteristics, such as small size, light weight, water- and moisture-proofing abilities, and multiplex networking capacities. By embedding FBG into flexible substrates with properties of high flexibility, high ductility, and free bending, flexible sensors were developed and extensively utilized in civil engineering, medical engineering, aerospace, and robotics etc. for the measurement of morphology, displacement, strain, and other measurands [1,2,3,4]. Recently, morphological sensing with FBG embedded flexible sensors has become a hot topic [5,6,7,8]. Payo et al. [9] measured the deflections of a robotic arm based on FBG sensors and interpolation methods. Xu et al. and Bhamber et al. [10,11] adopted orthogonal FBG arrays to measure bidirectional curvatures, then reconstructed shapes using a curve fitting algorithm. Yi et al. [12] studied a shape reconstruction method based on spatial movable coordinates, and realized the shape reconstruction of an aircraft frames. Kang et al. [13] modeled the relationship of wavelength–strain–displacement and reconstructed the entire structural deformations and local displacements of aluminum and acrylic beam specimens. Also, Todd et al. [14] employed a material-adapted reference frame together with a local linearization approach, for the purpose of reconstructing the shapes of slender. In the field of geotechnical engineering, researchers have studied the morphological sensing methods for measuring displacement profiles in critical areas of side slopes, subgrades, bridges, and other objects based on the FBG embedded flexible sensors. Li et al. [15] established a deflection–strain relationship for FBG flexible rods, which was then used in physical model tests to measure the displacements of the underground caves. Guo et al. [16] calculated the displacement profiles of beams using the strains and rotation angles measured by the FBG sensing points. Kim N.S. et al. [17] measured the deflection curves using a regression analysis method, and then applied the method for the measurement of bridge displacements. Xu et al. [18] estimated the bending deformations of the beams based on the curvature functions of cross-sections.

In practical applications, several factors may affect the measurement accuracy of morphology, including the layout intervals of FBG, the strain transferring rate from the substrate to FBG, the cumulative errors induced by algorithm etc. Therefore, it is critical to correct the reconstructed shapes using effective methods, and to improve the shape measurement accuracy. In terms of morphological sensing error analyses and corrections, Zhang et al. [19] proposed a dynamic error analysis method based on a least mean square algorithm. This method has been used for the morphological reconstructions and error analyses of space plate structures. Sun et al. [20] experimentally calibrated the relationship between the wavelength and bending curvatures, and improved the reconstruction precision of polyimide film deformations through the linear interpolation of the curvatures. Wang et al. [21,22] proposed an in situ calibrated deformation reconstruction method for FBG embedded geogrid, and improved the accuracy effectively. In the aforementioned research, unified correction coefficients were mainly applied to calibrate and correct the entire morphology of the structure to be measured, as well as to improve the precision of the morphological sensing. However, it has been found that, in practice, due to the diversity of the measured structural morphologies, the unified coefficients have been difficult to be applicable to a variety of morphologies, leading to major differences of the correction results for different shapes. While the shape correction methods based on the different morphological classifications for the FBG embedded flexible sensors have not yet been reported.

In this paper, a morphological sensing method, which is capable of correcting the reconstructed shape based on the morphological classification, was proposed to further improve the measurement accuracy of FBG implantable flexible morphological sensors. First, an FBG implantable flexible morphological sensor with temperature self-compensation capabilities was developed, and an arc curve fitting based on morphological sensing algorithm was introduced. Then, temperature calibration experiment indicated that the temperature self-compensation could be realized by the differences in the central wavelength variations of the two sensing points in the detecting unit. In the morphological calibration experiment, a conjugate gradient method was adopted to define the correction coefficients *k* of each morphology. A morphological sensing method which is capable of correcting the measured shapes based on different morphological classifications was proposed. The classification of different bending shapes was performed by using the extreme learning machine (ELM) algorithm. Finally, numerical simulations and experimental analyses were performed to verify the feasibility and effectiveness of the proposed method. The results indicate that the proposed method can improve the morphological measurement accuracy of FBG implantable flexible morphological sensors effectively, and it is of vital significance for the measurement of displacement fields in subgrades, bridges, and other geotechnical engineering applications.

## 2. Fabrication and Principle

### 2.1. Fabrication of the FBG Implantable Flexible Morphological Sensor

**Figure 1:**
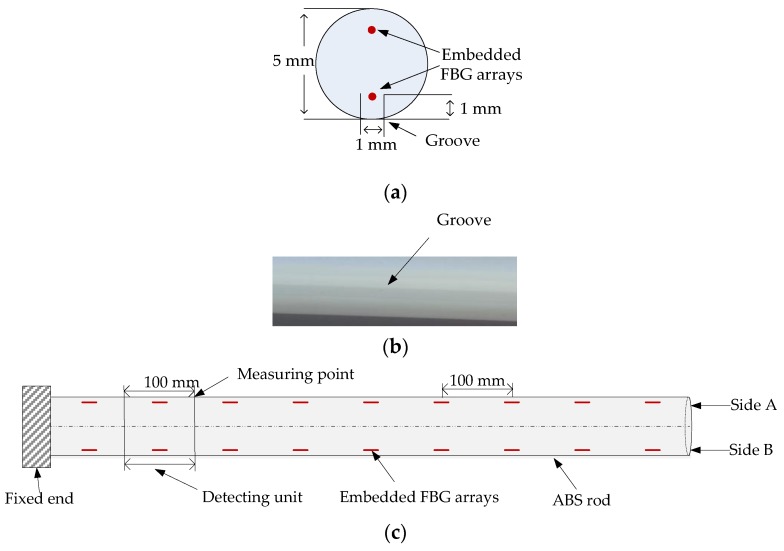
Schematic diagram of the proposed FBG implantable flexible morphological sensor. (**a**) Side view of sensor. (**b**) A groove on the surface of a flexible rod. (**c**) Positive view of the sensor.

**Figure 2:**
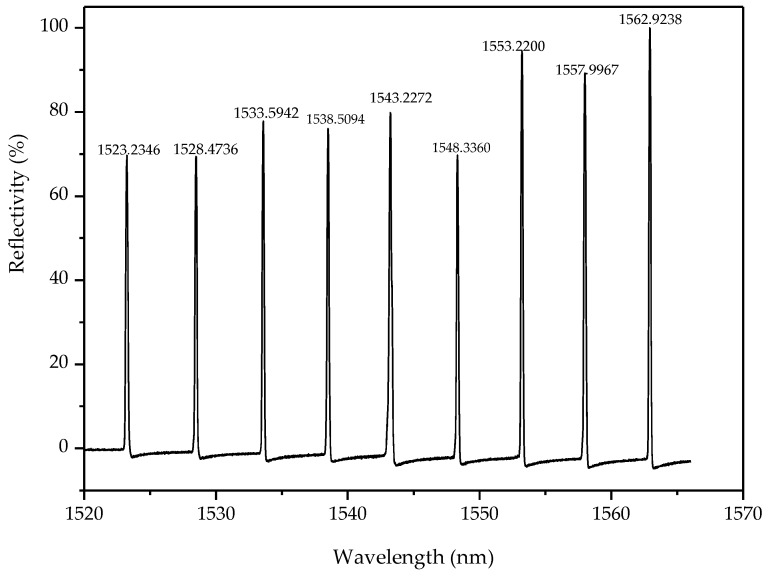
The schematic diagram of one FBG array full spectrum.

As detailed in Figure 1, for the proposed FBG implantable flexible morphological sensor, an acrylonitrile butadiene styrene (ABS) rod with high strength, corrosion resistance, and high temperature resistance as the flexible substrate. The flexible rod length was 900 mm, and the diameter was 5 mm. Two rectangular grooves (both depth and width: 1 mm) were slotted along the axis of the rod surface, and the interval of the two grooves was 180°. Plastic welding technology was used to implant two FBG sensing arrays into the two grooves for fixation purposes. FBG sensing arrays were fabricated using the phase mask method by Suzhou NanZee Sensing Technology Co., Ltd., Suzhou, China, of which each array had 9 FBG sensing points, and the spacing of two adjacent sensing points was 100 mm. The detailed parameters were shown in Table 1. An FBG demodulation instrument SM130 (MOI) was used to collect the central wavelength of the FBG sensing arrays, whose wavelength measurement ranged from 1510 nm to 1590 nm, and the demodulation precision was 1 pm. The full spectrum of the FBG array on one side in the free state was shown in Figure 2.

### 2.2. Morphological Sensing Model Based on Arc Curve Fitting Method

The FBG central wavelength is sensitive to axial strain and temperature, simultaneously [23,24]. The relationships between the central wavelength variations Δ*λ* and strain Δ*ε*, as well as the temperature Δ*T* are shown as follows:

(1) $$\frac{\Delta \lambda }{\lambda }=(\alpha_{f}+\xi )\Delta T+(1-P_{e})\Delta \varepsilon ,$$

where *λ* is the initial wavelength of FBG at free state, and *α_f_* and *ξ* is the thermal expansion coefficient and the thermal-optic coefficient of optical fiber, respectively. *P_e_* is the effective photo-elastic coefficient and is equal to 0.22.

The FBG sensing points divided the flexible rod into several microsegments, and each segment was taken as a detecting unit. The measuring points were located at the end of the detecting units, as detailed in Figure 1b. Then, in accordance with the principle of material mechanics [25,26], when the sensor was bent, the curvature *R_x_* of the detecting unit was

(2) $$R_{x}=\frac{\Delta \varepsilon_{x}}{Z},$$

where *Z* denoted the distance between the sensing points and the neutral axis, and Δ*ε_x_* represented the strain at the sensing points.

**Figure 3:**
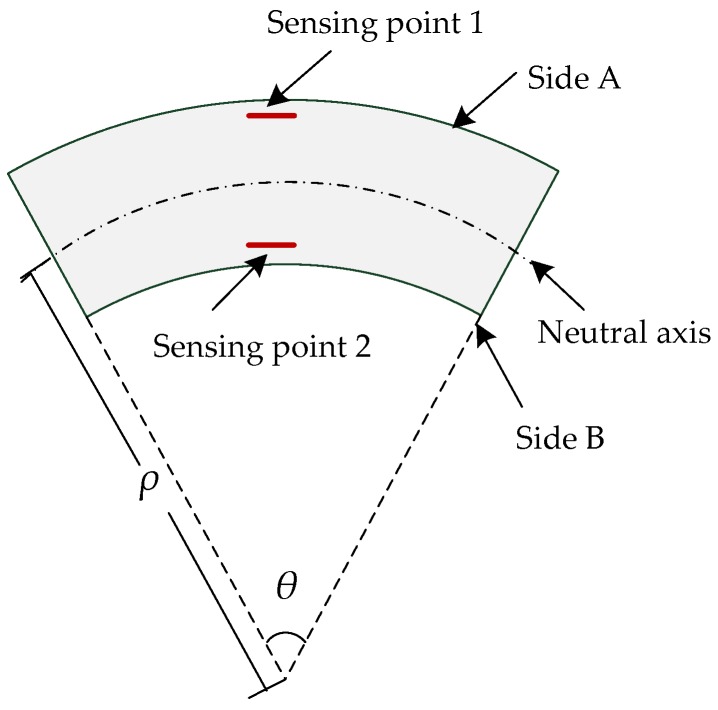
Measurement mechanism of each of the detecting unit.

In regard to the FBG sensing points arranged on the A and B symmetric sides of the detecting units, when the sensor rotated clockwise, sensing points 1 and 2 underwent tensile and compressive strain, respectively, as shown in Figure 3. Due to the fact that the two sensing points were equal distances from the neutral axis, the bending-induced wavelength variations were also equal in the opposite direction. Meanwhile, the temperature-induced wavelength variations displayed the same direction. When the detecting unit was bent, the strain of the detecting unit could be expressed as follows:

(3) $$\Delta \varepsilon_{x}=\frac{1}{2}(\Delta \varepsilon_{1}-\Delta \varepsilon_{2})=\frac{1}{2(1-P_{e})}(\frac{\Delta \lambda_{1}}{\lambda_{1}}-\frac{\Delta \lambda_{2}}{\lambda_{2}}).$$

As can be seen in Equations (2) and (3), the relationship between the curvature radius *ρ* of the detecting unit, and the central wavelength variation of the FBG were as follows:

(4) $$\rho =Z\cdot 2(1-P_{e})/(\frac{\Delta \lambda_{1}}{\lambda_{1}}-\frac{\Delta \lambda_{2}}{\lambda_{2}}).$$

Therefore, the curvature radius of the detecting unit could be obtained by acquiring the central wavelength variation of the FBG.

**Figure 4:**
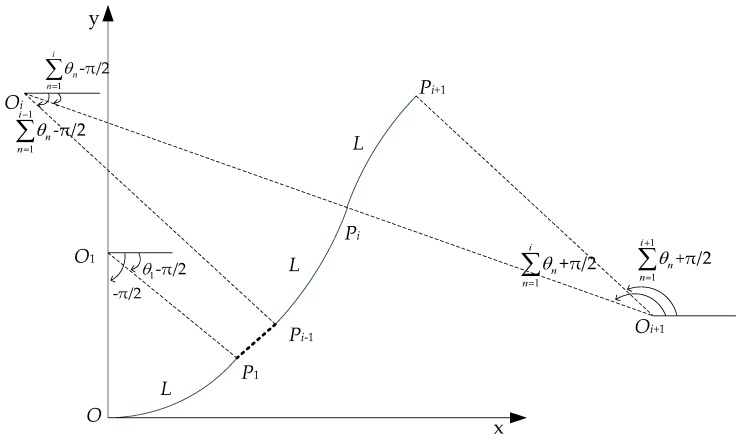
The schematic diagram of circular arc curve fitting method.

When the morphological sensor was bent, each detecting unit was continuous, in which the curvature radius *ρ* was greater than the arc length *L*. According to the differential principle, when the detecting unit length is small enough, the FBG implantable flexible morphological sensor can be regarded as being composed of many detecting units. Therefore, the curvature of the detecting unit at the FBG sensing point can be used to replace the curvature of the entire arc curve. For the convenience of calculation, it was stipulated in this study that when the curve was bent counterclockwise, then the curvature of the detecting unit was positive. Furthermore, when bending clockwise, the curvature was negative. Then, by assuming the starting point of the FBG implantable flexible morphological sensor was fixed with the coordinate of *O* (0, 0), each detecting unit could be considered as an arc microsegment. Following this, *O_i_*, *P_i_*, *ρ_i_*, and *θ_i_* represented the center, end point, bending radius, and center angle of the *i*-th arc segment, respectively. The angle *β_i_* of *P_i_* on the *i*-th arc curve was the angle between the *P_i_O_i_* and the horizontal line. When this was positive, the sensor was bent counterclockwise, while it was bent clockwise when it was negative, as shown in Figure 4.

In regard to the first arc curve *OP*_1_, the coordinate of the center *O*_1_ (*O*_1*x*_, *O*_1*y*_) was (0, *ρ*_1_), and the arc *OP*_1_ could be represented in rectangular coordinates as follows:

(5) $$\left\{ \begin{array}{l} x=O_{1x}+\rho_{1}\cdot \cos t\\ y=O_{1y}+\rho_{1}\cdot \sin t \end{array}\right.\;(-\pi /2\leq t\leq \theta_{1}-\pi /2).$$

When *t* = *θ*_1_ − *π*/2, the coordinate of point *P*_1_ (*P*_1*x*_, *P*_1*y*_) could be expressed as follows:

(6) $$\left\{ \begin{array}{l} P_{1x}=O_{1x}+\rho_{1}\cdot \sin\theta_{1}\\ P_{1y}=O_{1y}-\rho_{1}\cdot \cos\theta_{1} \end{array}\right.\;.$$

When *i* ≥ 2, the arc curve *P_i_*_−1_*P_i_* could be represented as follows:

(7) $$\left\{ \begin{array}{l} x=O_{ix}+\rho_{i}\cdot \cos t\\ y=O_{iy}+\rho_{i}\cdot \sin t \end{array}\right.\;(\sum_{n=1}^{i-1}\theta_{n}-\pi /2\leq t\leq \sum_{n=1}^{i}\theta_{n}-\pi /2).$$

Due to the tangency of the arc curves, the center *O_i_* of arc curve *P_i_*_−1_*P_i_*, center *O_i_*_+1_ of *P_i_P_i_*_+1_, and tangent point *P_i_* existed along the same straight line. When transiting from the curve *P_i_*_−1_*P_i_* to *P_i_P_i_*_+1_, the curvature changed from positive to negative. At this moment, the bending radius *ρ_i_*_+1_ and center angle *θ_i_*_+1_ of the curve *P_i_P_i_*_+1_ were negative. Then, the equation of the curve *P_i_P_i_*_+1_ could be expressed as follows:

(8) $$\left\{ \begin{array}{l} x=O_{(i+1)x}+(-\rho_{i+1})\cdot \cos t\\ y=O_{(i+1)y}+(-\rho_{i+1})\cdot \sin t \end{array}\right.\;(\sum_{n=1}^{i+1}\theta_{n}+\pi /2\leq t\leq \sum_{n=1}^{i}\theta_{n}+\pi /2).$$

The angle *β_i_* of point *P_i_* on the arc curve *P_i_*_−1_*P_i_* was $\sum_{n=1}^{i}\theta_{n}-\pi /2$, and the angle *β_i_*_+1_ on curve *P_i_P_i_*_+1_ was $\sum_{n=1}^{i}\theta_{n}+\pi /2$. Then, in accordance with Equations (6)–(8), the measuring point coordinate *P_i_* (*P_ix_*, *P_iy_*) at the end of each arc curve was as follows:

when *i* = 1,

(9) $$\left\{ \begin{array}{l} P_{1x}=O_{1x}+\rho_{1}\cdot \sin\theta_{1}\\ P_{1y}=O_{1y}-\rho_{1}\cdot \cos\theta_{1} \end{array}\right.\;;$$

when *i* ≥ 2,

(10) $$\left\{ \begin{array}{l} P_{ix}=O_{(i-1)x}+(\rho_{\left(i-1\right)}-\rho_{i})\cdot \sin\sum_{n=1}^{i-1}\theta_{n}+\rho_{i}\cdot \sin\sum_{n=1}^{i}\theta_{n}\\ P_{i}{}_{y}=O_{(i-1)y}-(\rho_{\left(i-1\right)}-\rho_{i})\cdot \cos\sum_{n=1}^{i-1}\theta_{n}-\rho_{i}\cdot \cos\sum_{n=1}^{i}\theta_{n} \end{array}\right..$$

## 3. Calibration Experiments

### 3.1. Temperature Calibration Experiment

In order to avoid the influences of temperature on the morphological sensing precision, a temperature calibration experiment was first carried out to verify the temperature characteristics and self-compensation effects of the FBG implantable flexible morphology sensor. The FBG implantable flexible morphology sensor was placed in the calorstat, with the temperature gradually adjusted from −20 °C to 40 °C. The temperature was raised by 10 °C each time, and the calorstat temperature measurement precision was 0.01 °C. SM130 was used to collect the central wavelengths of the two FBG arrays. 

**Figure 5:**
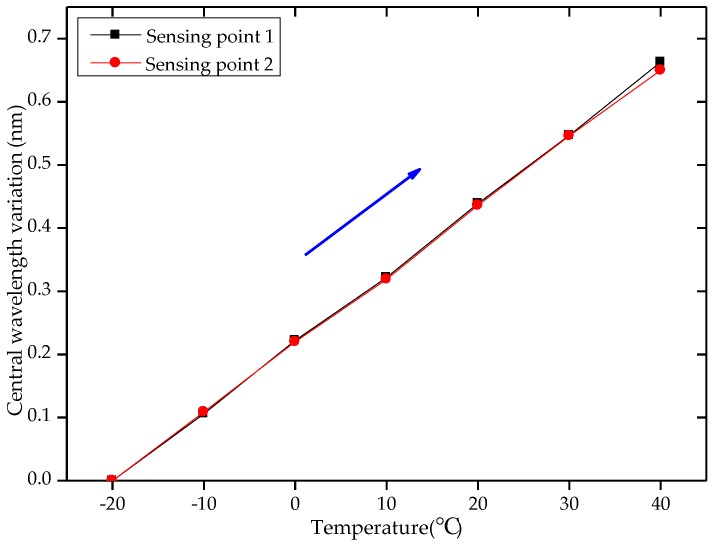
Temperature response characteristics curves of two sensing points.

The temperature response coefficients of the two FBG arrays were shown in Table 2. It can be seen in Table 2 that the temperature response coefficients of the sensing points in the same detecting unit were basically the same. The temperature response curves of two sensing points in one of the detecting units are shown in Figure 5. Sensing point 1 and sensing point 2 were implanted on the sides of A and B, respectively, in which the relationships between the central wavelength variation and the temperature variation were *λ*_1_ = 0.0110Δ*T* + 0.2181 and *λ*_2_ = 0.0109Δ*T* + 0.2171, respectively. Therefore, the temperature self-compensation could be realized by the differences in the central wavelength variations of the two sensing points in the detecting unit.

### 3.2. Morphological Calibration Experiment

**Figure 6:**
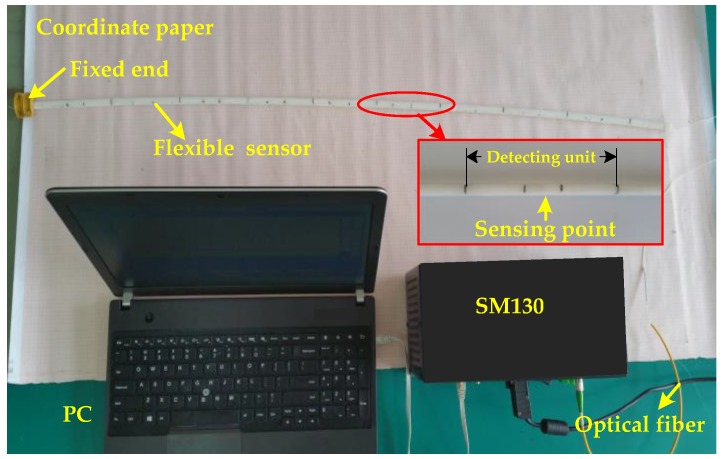
Schematic diagram of experimental facility.

**Figure 7:**
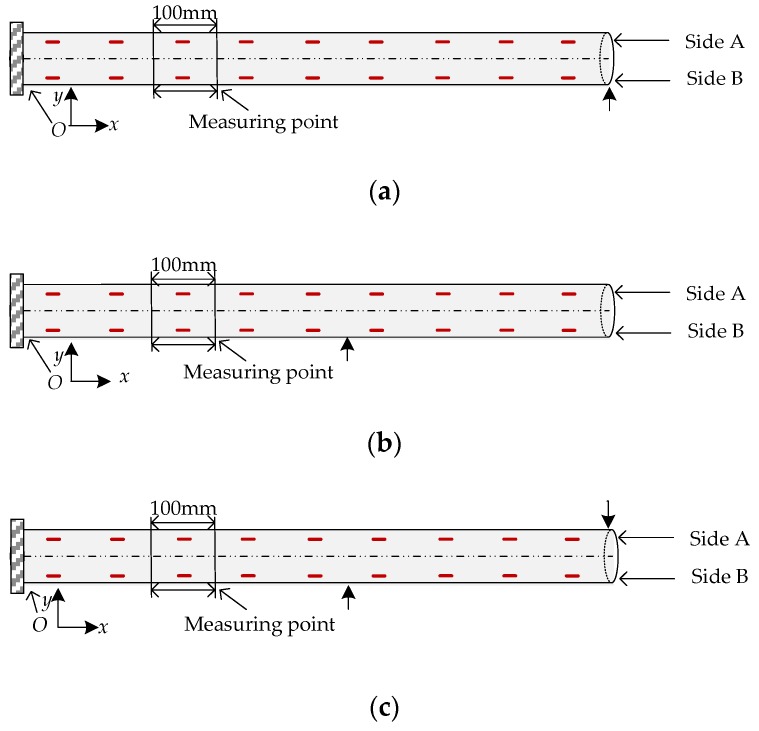
Schematic diagram of the bending shapes of the FBG implantable flexible morphological sensor under different loading modes. (**a**) Type 1: applying force at the end of the sensor. (**b**) Type 2: applying force at the middle of the sensor. (**c**) Type 3: applying force at two points of the sensor.

A morphological bending experiment was carried out in this study in order to verify the feasibility of the morphological classification correction method, as shown in Figure 6. One end of the FBG implantable flexible morphological sensor was fixed on the calibration platform with coordinate paper (cell: mm^2^). The coordinate of the fixed point on side B was *O* (0, 0), and the coordinates of each measuring point on side B were *P_j_* (100*j, 0), with *j* = 1, 2, … 9 (see Figure 7). On the other end of the sensor, the tail fiber was connected to the SM130, and the wavelength data of the two FBG arrays obtained by the SM130 demodulation were transmitted to the computer via a network cable. Then, displacements were applied to the different locations of the sensor for the formation of the different bending shapes, and the displacements of each measuring point were read by a high-precision displacement sensor (precision: 0.01 mm).

The experiment was conducted on three typical bending shapes of the FBG implantable flexible morphological sensors, as shown in Figure 7a–c, and six different sizes of displacement were applied to each type. For Type 1 (Figure 7a), six different sizes of displacement were applied to the sensor at (900, 0) along the *y* direction, at 80 mm, 142 mm, 215 mm, 282 mm, 347 mm, and 400 mm, respectively. For Type 2 (Figure 7b), the displacements were applied along the *y*-axis at (500, 0) at 70 mm, 123 mm, 142 mm, 157 mm, 181 mm, and 211 mm, respectively. For Type 3 (Figure 7c), the displacements were applied to the sensor at (500, 0) and (900, 0) along the *y* direction, respectively, and the displacement values in the six cases were 50 mm/17 mm, 97 mm/49 mm, 96 mm/42 mm, 121 mm/63 mm, 151 mm/71 mm, and 190 mm/165 mm, respectively. In each case, ten repetitions were performed. Then, SM130 was used to collect the central wavelength of the FBG sensing points. When the displacement was determined to be stable, the real displacement values of the measuring points were read by a high-precision displacement sensor. By using the actual displacement values of the measuring points as the benchmark, the optimization goal was to minimize the mean relative errors (MREs) between the reconstructed displacement and real displacement at each measuring point. The calculation formula of the MRE was $\frac{1}{n}\sum_{i=1}^{n}\left|y_{Ci}(k)-y_{Ai}\right|$, where *n* = 9, $y_{Ci}(k)$ was the calculation displacement of each measuring point based on corrected strain value *k* *Δ*ε_x_* of each detecting unit and arc curve fitting method, and *y_Ai_* was the real displacement of each measuring point. Then, a conjugate gradient method was adopted to define the correction coefficients *k* of each morphology. Conjugate gradient method has the characteristics of simple iterative format, small amounts of storage required, high stability and fast convergence speed, which can be used to solve optimization problems. A detailed formula description of conjugate gradient method was shown as follows.

The optimization problem was

(11) $$\min f(k)=\frac{1}{n}\sum_{i=1}^{n}\left|y_{Ci}(k)-y_{Ai}\right|.$$

The sequence {*k_m_*} could be obtained by the following iterative format,

(12) $$k_{m+1}=k_{m}+\alpha_{m}d_{m},$$

where *d_m_* could be expressed as follows:

(13) $$d_{m}=\left\{ \begin{array}{ll} -g(k_{m}), & m=0\\ -g_{m}(k_{m})+\beta_{m}d_{m-1}, & m\geq 1 \end{array}\right.,$$

where

(14) $$g(k_{m})=\nabla f(k_{m}).$$

$\alpha_{m}$ was search step size, which obtained by satisfying the following weak Wolfe conditions,

(15) $$f(k_{m}+\alpha_{m}d_{m})-f(k_{m})\leq \delta \alpha_{m}g{\left(k_{m}\right)}^{T}d_{m},$$

(16) $$g{\left(k_{m}{}_{+1}\right)}^{T}d_{m}\geq \sigma g{\left(k_{m}\right)}^{T}d_{m},$$

where 0<δ≤σ<1. $\beta_{m}$ was the scalar of the representation algorithm and PRP method was selected in this paper, which could be expressed as follows:

(17) $$\beta_{m}^{PRP}=g{\left(k_{m}\right)}^{T}f(k_{m-1})/{\left\|g(k_{m-1})\right\|}^{2}.$$

The correction coefficient *k* of each morphology was obtained by the conjugate gradient method (see Table 3). Correction coefficients for Types 1 and 2 were determined to be *k*_1_ = 1.45 and *k*_2_ = 1.80, respectively. For Types 3, the correction coefficients of the positive and negative strain values were *k*_3_^+^ = 1.49 and *k*_3_^−^ = 1.10, respectively. The unified correction coefficient was the average of *k*_1_, *k*_2_, *k*_3_^+^, and *k*_3_^−^, and denoted as $\bar{k}$= 1.46. However, no other morphological experiments were carried out in this study, due to the limitations of the yield strength of the flexible substrate, experimental conditions, and other factors. However, there were still sufficient results achieved to confirm the feasibility of the proposed correction method. 

## 4. Correction Methods

The morphological sensing algorithm which was based on arc curve fitting has the ability to evaluate the shape of the FBG implantable flexible morphological sensor. Thus, it is able to evaluate the overall displacement profiles of the structures to be measured. However, the following factors may cause large morphological sensing errors and affect the precision of the displacement measurements. First, during the sensor fabrication processes, curvature measurement errors at the FBG sensing points may easily be caused due to the deviations when implanting the FBG into the substrate. Second, the curvatures measured by the flexible sensor are discontinuous, which is caused by a certain interval between the adjacent FBG sensing points. The curvatures of the FBG sensing points are used to replace the curvatures of the detecting units in the calculation process of the arc curve fitting algorithm, which may induce the measurement errors. Third, the morphological sensing method is the integration of the piece-wise detecting units, and the displacement errors of each segment will gradually accumulate to generate accumulated errors at the end of the sensor.

To further improve the measurement accuracy of the FBG implantable flexible morphological sensor, the different bending shapes of the sensor were automatically categorized using the intelligent classification algorithm. By calibrating the typical bending shapes of the sensor, the correction coefficients of different shapes were determined, and the corresponding correction coefficients of different bending shapes was then selected automatically to correct the reconstructed shapes for the purpose of minimizing the errors. 

ELM algorithm was adopted to intelligently classify the bending shapes of the FBG implantable flexible morphological sensor in this research study. ELM is a novel intelligence optimization algorithm for single-hidden layer feedforward neural networks (SLFNs), which was proposed in 2004 [27]. This algorithm randomly selects hidden nodes and analytically determines the output weights of SLFNs. Compared with conventional popular learning algorithms, this algorithm has the characteristics of extremely fast learning speed, good generalization performance, and tuning of free parameters. A detailed formula description of ELM was presented in Ref. [28]. 

Define that there are *N* arbitrary distinct training samples $(x_{i},y_{i})\in R^{n}\times R^{m}$, where *x* are the training inputs and *y* are the training targets. The function of ELM classifier with *L* hidden layer neurons and a sigmoid function *g*(*x*) is mathematically modeled as follows:

(18) $$\sum_{j=1}^{L}\beta_{j}g(a_{j},b_{j},x_{i})=y_{i},\;i=1,2,\ldots ,N.,$$

where $\beta_{j}$ is output weight matrix, $a_{j}$ is input weight matrix, $b_{j}$ is bias connecting the input neurons.

According to any continuous probability distribution, $a_{j}$,$b_{j}$ can be randomly generated. Therefore, the Equation (18) can be written in the form of the following matrix.

(19) Hβ=Y,

where

(20) $$H=[\begin{array}{ccc} g(a_{1},b_{1},x_{1}) & \cdots & g(a_{L},b_{L},x_{1})\\ \vdots & \ddots & \vdots \\ g(a_{1},b_{1},x_{N}) & \cdots & g(a_{L},b_{L},x_{L}) \end{array}{]}_{N\times L},$$

(21) $$\beta ={\left[\begin{array}{c} \beta_{1}^{Y}\\ \;\vdots \\ \beta_{L}^{Y} \end{array}\right]}_{L\times m}\;\mathrm{and}\;Y={\left[\begin{array}{c} y_{1}^{Y}\\ \;\vdots \\ y_{N}^{Y} \end{array}\right]}_{N\times m},$$

where H is the hidden layer output matrix of ELM. 

These training algorithms need to adjust the input weights and the hidden layer biases, and the output weights can be given as follows:

(22) $$\beta =H^{†}Y,$$

where $H^{†}$ is the Moore–Penrose generalized inverse of matrix H.

When $H^{T}H$ is nonsingular,

(23) $$H^{†}={\left(H^{T}H\right)}^{-1}H^{T},$$

or when $HH^{T}$ is nonsingular,

(24) $$H^{†}=H^{T}{\left(HH^{T}\right)}^{-1}.$$

Therefore, the output function of ELM classifier can be shown as follows:

(25) $$f(x)=h(x)H^{†}Y.$$

The steps of classification of the morphological correction method were presented as follows:

**Step 1**: The central wavelengths of the two FBG arrays were obtained using an FBG demodulator, and Equation (4) was used to convert the wavelength variations at the sensing point to the curvature of detecting unit. Then, based on an arc curve fitting method, the bending shapes of the FBG implantable flexible morphological sensor were first reconstructed.

**Step 2**: The sensor was calibrated in order to obtain the actual displacement of each measuring point in the typical bending shape. Then, a conjugate gradient method was applied to determine the correction coefficients *k* of the different bending shapes, with the actual displacement as the standard.

**Step 3**: The experimental process was completed on different bending shapes, and then Step 1 was repeated. The displacement of the measuring point *P_i_* along the *y*-axis was taken as the input. Meanwhile, different bending shape types were regarded as the output in order to train the ELM classifier model.

**Figure 8:**
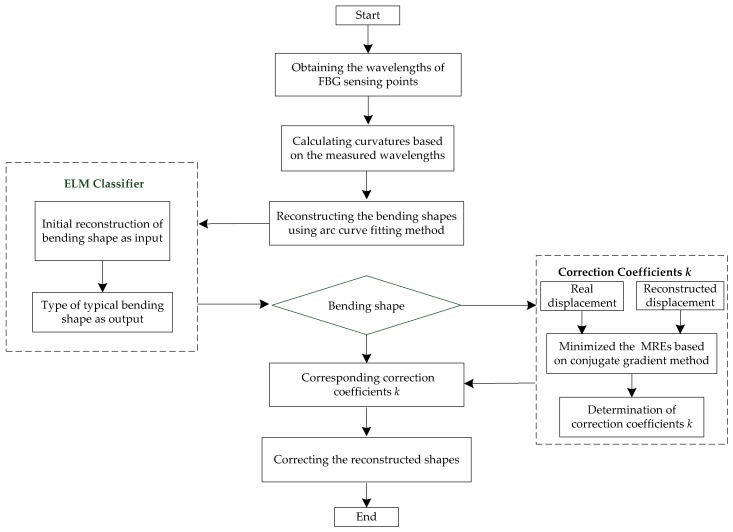
Flow chart of classification morphological correction method based on ELM intelligent algorithm.

**Step 4**: An ELM classifier was used to classify the bending shapes first reconstructed in Step 1. Then, the corrected strain value *k* *Δ*ε_x_* of each detecting unit determined in Step 2 was applied to reconstruct the bending shapes of the FBG implantable flexible morphological sensor in order to obtain an accurate bending shape. The specific process was shown in Figure 8.

The above method was adopted for bending shape classification correction of the proposed FBG implantable flexible morphological sensor, which had the ability to effectively reduce the displacement errors of the measuring points caused by the sensor preparation errors, FBG network capacity limitations, and other factors. Therefore, the classification morphological correction method proposed in this study displayed the ability to improve the intellisense displacement abilities of the FBG flexible morphological sensor.

## 5. Simulation Verification

**Figure 9:**
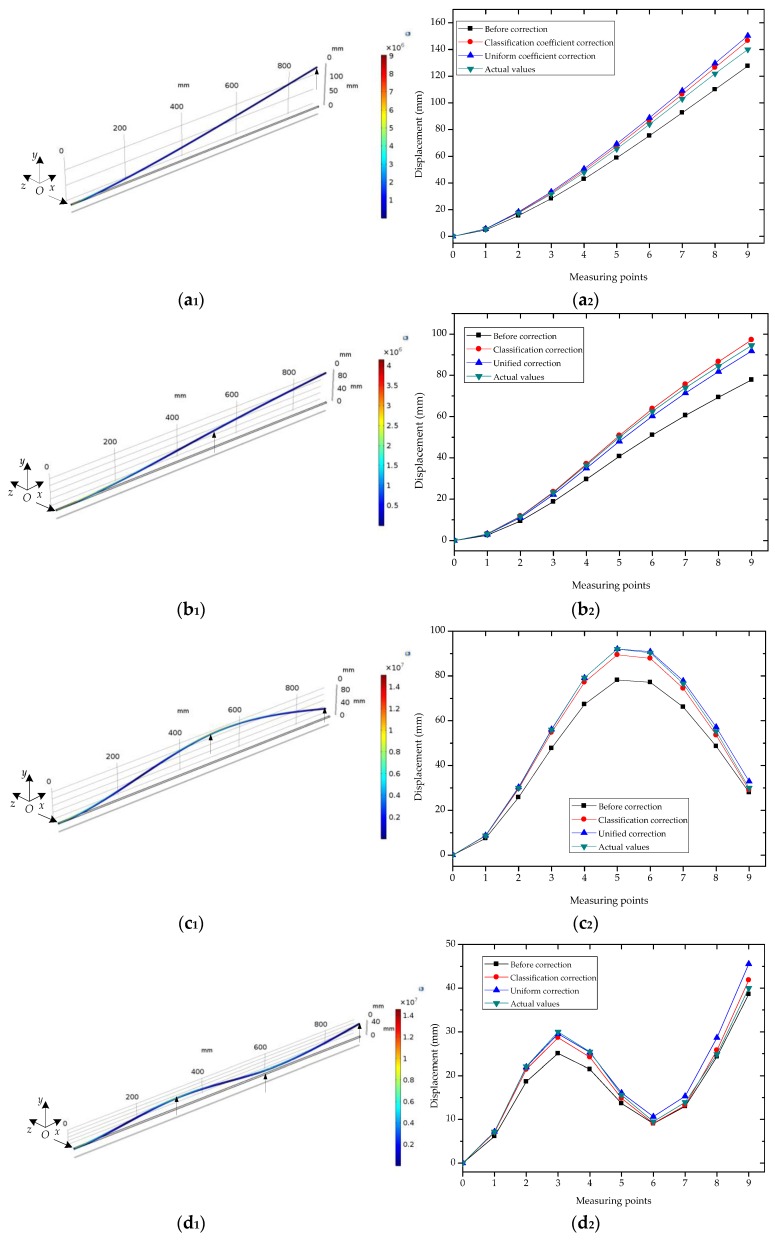

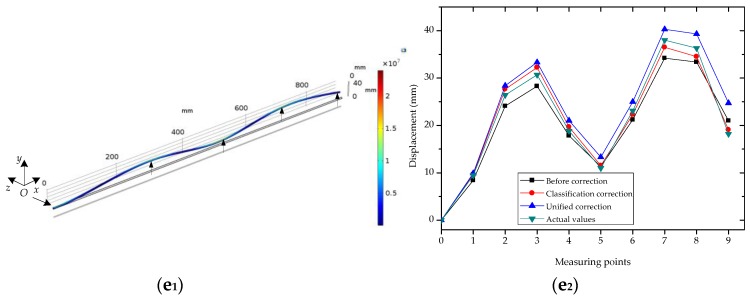
The simulation diagram of the bending shapes of the FBG implantable flexible morphological sensor under different loading modes and displacement at measuring points were obtained based on the arc curve fitting algorithm. (**a_1_**) Simulation of applying force at the end of the model. (**a_2_**) Displacement comparison of measuring points at Type 1. (**b_1_**) Simulation of applying force at the middle of the model. (**b_2_**) Displacement comparison of measuring points at Type 2. (**c_1_**) Simulation of applying force at two points of the model. (**c_2_**) Displacement comparison of measuring points at Type 3. (**d_1_**) Simulation of applying force at three points of the model. (**d_2_**) Displacement comparison of measuring points at Type 4. (**e_1_**) Simulation of applying force at four points of the model. (**e_2_**) Displacement comparison of measuring points at Type 5.

In this study, a finite element method was applied to simulate the bending shapes of the FBG implantable flexible morphological sensor, and to compare the morphological sensing effects which had been obtained by the unified coefficient and classification coefficient corrections. First, a cylinder model with a length of 900 mm and a diameter of 5 mm was established, as shown in Figure 9a_1_,b_1_,c_1_,d_1_,e_1_. The initial end center coordinates of the cylinder model was *O* (0, 0, 0), and the cylinder model was divided into nine segments along the *x*-axis. Each segment was taken as a detecting unit of the proposed FBG implantable flexible morphological sensor. The column material was ABS, and its mechanical property parameters were shown in Table 4. In this study’s simulation, the cylinder model had a total of 4379 meshes with minimum unit masses of 0.351. Then, with consideration given to the geometrical nonlinearity of the material used in the simulation, an elastic model was selected.

A displacement constraint was fixed on one side of the cylinder model, and five different types of displacement were applied in order to simulate five different typical bending shapes. For Type 1 (Figure 9a_1_), a 140 mm displacement was applied along the *y*-axis at (900, −2.5, 0) in the cylinder model. For Type 2 (Figure 9b_1_), a 50 mm displacement was applied along the *y*-axis at (500, −2.5, 0). For Type 3 (Figure 9c_1_), 92 mm and 30 mm displacements were applied along the *y*-axis at (500, −2.5, 0) and (900, −2.5, 0), respectively. For Type 4 (Figure 9d_1_), 30 mm, 9.5 mm, and 40 mm displacements were applied along the *y*-axis at (300, −2.5, 0), (600, −2.5, 0), and (900, −2.5, 0), respectively. For Type 5 (Figure 9e_1_), 30.7 mm, 11 mm, 38 mm, and 18.2 mm displacements were applied along the *y*-axis at (300, −2.5, 0), (500, −2.5, 0), (700, −2.5, 0), and (900, −2.5, 0), respectively. For the five typical bending shapes, the strain value at *i* = 0, 1, ... 8 in the coordinate points of *S_i_*(50 + 100*i, −2.5, 0) were extracted from the cylinder model. Meanwhile, the displacement at *j* = 1, 2, ... 9 in the coordinate points of *P_j_* (100*j, −2.5, 0) were extracted as the standard displacement values. The strain values which had been extracted were used for the first reconstruction of the different bending shapes of the cylinder model by arc curve fitting method, in order to obtain the displacement of the measuring points under different bending shapes, as shown in Figure 9a_2_,b_2_,c_2_,d_2_,e_2_. It was observed that, having been influenced by multiple factors (such as the accumulated errors of detecting units), there were certain errors between the first reconstructed displacement and the simulated displacement. For Types 1 and 2, the relative errors of the measuring points away from the fixed end had gradually increased (with maximum errors of 12.36 mm and 16.66 mm, respectively), and both were located at the ninth measuring point. This was determined to be due to the fact that the cylinder model had undergone uniaxial stress conditions for Types 1 and 2, and each measuring point displayed the same displacement error direction. The displacement errors of the measuring points tended to accumulate point by point. However, for Types 3, 4, and 5, the measuring points with the maximum relative errors were located in the fifth, third, and seventh points, with relative error values of 13.81 mm, 4.93 mm, and 3.79 mm, respectively. These results may be due to the fact that the cylinder model had undergone multidirectional stress conditions for Types 3, 4, and 5, and the displacement errors of the measuring points displayed the phenomena of positive and negative error offsets.

**Figure 10:**
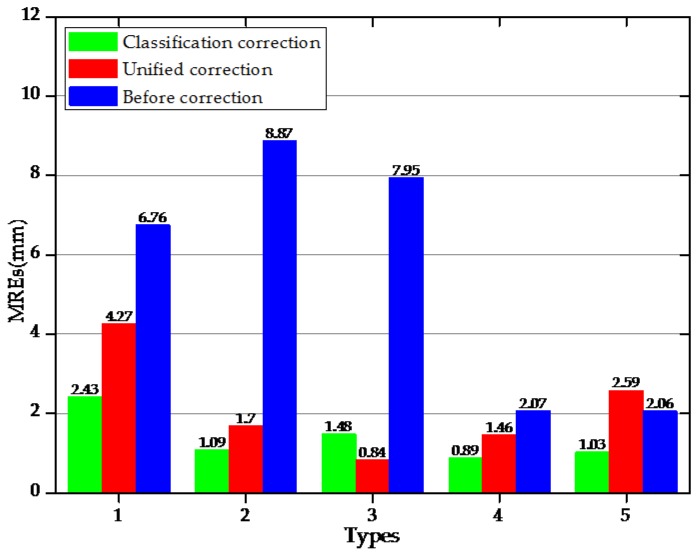
Comparison of MREs based on different correction methods under five bending shapes.

During this study’s experimental process, based on the actual applied displacement as the benchmark, weighted corrections were conducted on the first reconstructed morphology. Then, a conjugate gradient method was adopted to define the correction coefficient of each morphology. As detailed in Table 5, correction coefficients for Types 1 and 2 were determined to be *k*_1_ = 1.15 and *k*_2_ = 1.25, respectively. For Types 3, 4, and 5, the correction coefficients of the extracted positive and negative strain values were *k*_3_^+^ = 1.15 and *k*_3_^−^ = 1.17, *k*_4_^+^ = 1.14, *k*_4_^−^ = 1.18, *k*_5_^+^ = 1.19, *k*_5_^−^ = 1.21, respectively. The unified correction coefficient was the average of *k*_1_, *k*_2_, *k*_3_^+^, *k*_3_^−^, *k*_4_^+^, *k*_4_^−^, *k*_5_^+^, and *k*_5_^−^, and denoted as $\bar{k}$= 1.18. Figure 10 shows the MREs of the sensing displacement of the cylindrical model under the five bending shapes based on different correction methods. It was observed that under the different bending shapes, the MREs of the measuring points were significantly different when the strain values in the center of the detecting units were corrected using the unified coefficients. For Types 1, 2, 3, and 4, the MREs of the corrected measuring points were determined to decline, of which that of Type 3 displayed the minimum decline of 0.84 mm, while that of Type 1 displayed the maximum decline of 4.27 mm. For Type 5, the MREs had increased following the corrections of the unified coefficients. However, for the different bending shapes, the errors in the measuring points which had been corrected by the different coefficients had obviously decreased. The MREs had been reduced by 4.33 mm (Type 1), 7.78 mm (Type 2), 6.47 mm (Type 3), 1.18 mm (Type 4), and 1.03 mm (Type 5), respectively, after the classification corrections. These findings indicated that, when compared with the unified coefficient corrections, the classification corrections of the different bending shapes had improved the measurement precision of the displacements, which confirmed that it was necessary to use different coefficient corrections for the various bending shapes.

## 6. Experimental Analysis

### 6.1. Morphological Classification Based on ELM Algorithm

**Figure 11:**
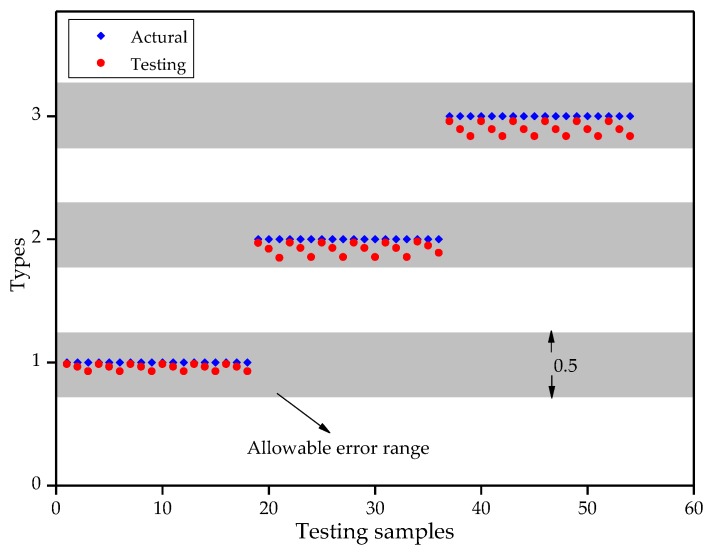
Classification results of bending shapes based on testing samples.

In Section 3.2, morphological calibration experiment was conducted on three typical bending shapes of the FBG implantable flexible morphological sensors. Six different sizes of displacement were applied to each type, and ten repetitions were performed in each case. An arc curve fitting algorithm was used to calculate the initial morphology of the FBG implantable flexible morphological sensor. On this basis, an ELM algorithm was adopted to train the displacement data in the initial morphology along the *y* direction. Also, the different bending shapes were intelligently classified. To be specific, for the six circumstances of each bending shape, the repetitive experimental data of the previous seven times of each circumstance were taken as the training samples (training samples totaled 126 groups). The displacement data of the training sample along the *y*-axis were used as the input, while the bending shape Types (Types 1, 2, and 3) were adopted as the output, in order to establish an ELM model. The repetitive experimental data of the later three times were taken as the test samples (test samples totaled 54 groups) in order to verify the classification effects, and the results are shown in Figure 11. The classification intervals of Types 1, 2, and 3 were [0.75, 1.25], [1.75, 2.25], and [2.75, 3.25], respectively. It was observed that for the 54 groups of test sample data, the ELM algorithm was able to effectively realize the intelligent classification of the three typical bending shapes of the FBG implantable flexible morphological sensor. The ELM algorithm was implemented based on MATLA programming software, with an entire program running time of 477 ms (computer configuration: Inter(R) Core(TM) i5-4210M CPU and 4G RAM). The results indicated that the algorithm had a fast calculation speed with good generalization ability, and was able to effectively ensure the real-time performance of the morphological sensing.

### 6.2. Comparison of Morphological Sensing Based on Different Correction Methods

In this study, for each type of bending shape, the repetitive experimental data of the previous three times in one experimental circumstance were selected to compare the effects from the unified coefficient correction and the classification coefficient correction on the displacement sensing of the FBG implantable flexible morphological sensor. For each group of experimental data, the classification morphological correction method and an arc curve fitting algorithm were used to calculate the displacement of each measuring point, respectively. The standard deviations of the displacements at each measuring point for each bending shape are shown in Table 6.

**Figure 12:**
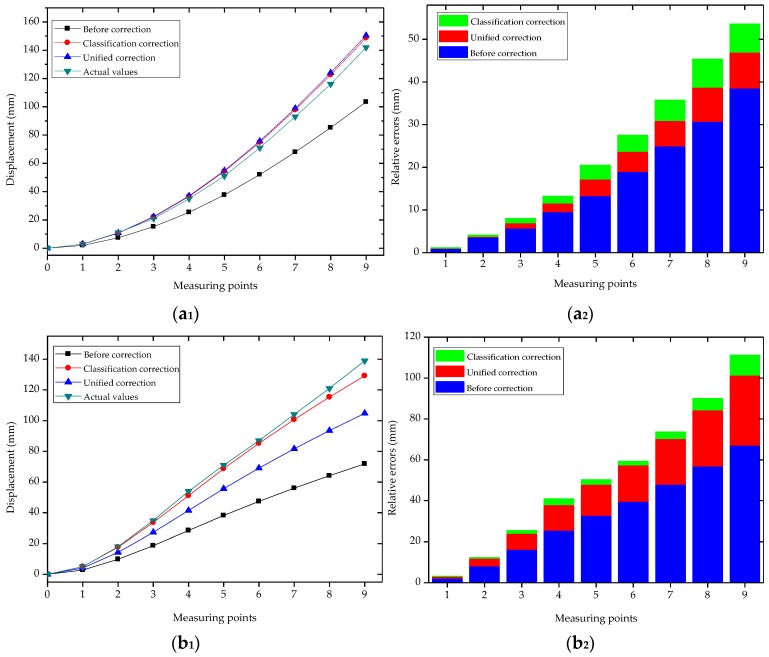

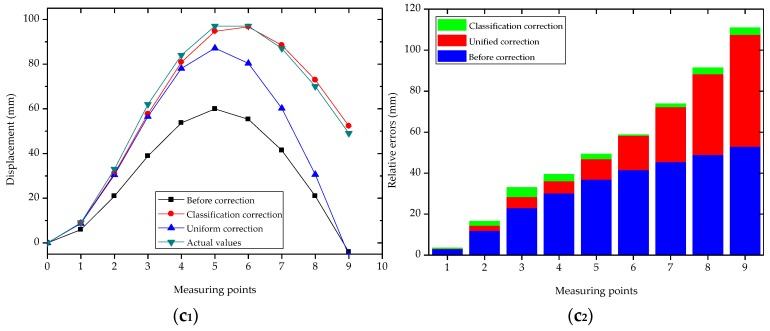
Demonstration of experimental results under three bending shapes of FBG implantable flexible morphological sensor. (**a_1_**) Displacement comparison of measuring points at Type 1. (**a_2_**) Relative errors comparison of measuring points at Type 1. (**b_1_**) Displacement comparison of measuring points at Type 2. (**b_2_**) Relative errors comparison of measuring points at Type 2. (**c_1_**) Displacement comparison of measuring points at Type 3. (**c_2_**) Relative errors comparison of measuring points at Type 3.

**Figure 13:**
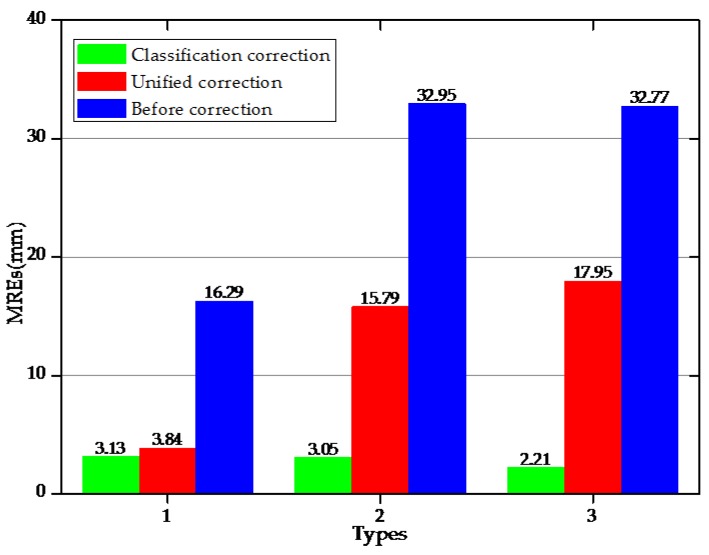
Comparison of mean relative errors (MREs) based on different methods under three bending shapes.

For each bending shape, the average of repetitive experimental data of the previous three times was taken. Figure 12 shown the contrast between the displacements of the measuring points before and after the corrections and the real displacements Figure 12a_1_,b_1_,c_1_ under the different bending shapes, as well as the relative errors of the measuring points before and after the corrections Figure 12a_2_,b_2_,c_2_. It can be seen in Figure 12a_2_,b_2_,c_2_ that there were certain errors between the first reconstructed displacements and the real displacements. It was observed that among these, with the increasing distances between the measuring points and fixed point, the relative errors of Types 1, 2, and 3 had gradually increased. The maximum relative errors had all occurred to the ninth measuring point (for example: 38.57 mm, 67.15 mm, and 53.06 mm). It can be seen that the first reconstructed displacement of the measuring point had a larger error. Following the corrections, the errors of the three bending shapes had been significantly reduced. The MREs obtained by the first reconstruction, along with the different correction methods, were shown in Figure 13. After the central strain values of detecting units were corrected by the unified coefficients, the MREs of the measuring points displayed larger differences. For example, for Type 1, the MRE of the measuring points after correction was the minimum at 3.84 mm; for Type 2, the MRE was 15.79 mm; for Type 3, the MRE was the maximum at 17.95 mm. However, for the different bending shapes with different coefficient corrections, it could be seen that the MREs of measuring points had obviously decreased. The MREs of Types 1, 2, and 3 after the classification corrections were 4.8 mm, 1.67 mm, and 2.4 mm, respectively. The MREs had been reduced by 13.16 mm (Type 1), 29.90 mm (Type 2), and 30.56 mm (Type 3), respectively, following the classification corrections. When compared with the unified coefficient corrections, the classification coefficient corrections displayed a good correction ability for the displacement errors of the measuring points with different bending shapes.

Table 7 shows the relative error percentages at each measuring point under the three bending shapes following the classification corrections. It could be seen that under the different bending shapes, the maximum relative error percentages of each measuring point of the FBG implantable flexible morphological sensor were 6.39% (Type 1), 7.04% (Type 2), and 7.02% (Type 3), respectively. Following the corrections of the different bending shapes, the relative error percentages of each measuring point were observed to be relatively low. These findings indicated that the proposed classification morphological correction method was feasible, and the method was found to effective in improving the precision of the FBG implantable flexible morphological sensor when sensing deformation fields in geotechnical engineering related applications.

## 7. Conclusions

In this research, an FBG implantable flexible morphological sensor with the capacity of temperature self-compensation, bending shape self-classification, and displacement error self-correction was designed by internally implanting a 180° spacing FBG array in an ABS rod. On this basis, the causes of the morphological sensing errors were analyzed for an FBG implantable flexible morphological sensor. Meanwhile, the key focus was to propose a morphological sensing method for an FBG implantable flexible sensor based on classification morphological corrections. A temperature calibration experiment was performed to verify that the influences of temperature could be eliminated by using the central wavelength variations of two FBG sensing points in each detecting unit. Although limited by such factors as the yielding strength of the flexible rod substrate and various experimental conditions, this study’s experiments were carried out on three typical bending shapes of the FBG implantable flexible morphological sensor, and the correction coefficients *k* of each morphology were obtained by the conjugate gradient method. A finite element simulation was adopted for five typical bending shapes of a cylinder model, and the necessity and feasibility of the proposed method were analyzed. The intelligent classification of the different bending shapes were realized using an ELM classifier. During this study’s experimental process, the MREs of the measuring points were observed to be significantly reduced following the coefficient corrections of the different bending shapes. The MREs were found to be reduced by 13.16 mm (Type 1), 29.90 mm (Type 2), and 30.56 mm (Type 3), respectively, following the classification corrections. When compared with the unified coefficient corrections, the classification coefficient corrections displayed a good correction ability for the displacement errors at the measuring points in the different bending shapes. Following the corrections by the classification morphological correction method, the maximum relative error percentages of the displacements at measuring points in different bending shapes were determined to be 6.39% (Type 1), 7.04% (Type 2), and 7.02% (Type 3), respectively. Following the corrections of the different bending shapes, the relative error percentages of each of the measuring points were relatively low. These findings confirmed that the proposed classification correction method was feasible and effective in improving the intelligent displacement sensing ability of the FBG implantable flexible morphological sensor. However, due to the limitations of the experimental conditions and the properties of the substrate material, the experimental validation was only carried out on three morphologies in this study. In the future, the correction performances of various bending shapes will be considered in detail, and the effects of the proposed classification morphological correction method under three-dimensional coordinates will be further examined. This study’s designed FBG implantable flexible morphological sensor can be potentially used for the real-time monitoring of deformation fields in rock masses, subgrades, bridges, as well as other geotechnical engineering processes, and presents vital significance and application promotion value. 

## Figures and Tables

**Table 1:** The detailed parameters of fiber Bragg grating (FBG) sensing arrays.

Parameters	Description
fiber type	single mode fiber
grating type	9-point string
grating length	10mm
bandwidth 3 dB	<0.2nm
side lobe suppression ratio	>15dB

**Table 2:** Temperature response coefficients at each sensing point (SP).

Position	Temperature Response Coefficients (nm/°C)								
	SP 1	SP 2	SP 3	SP 4	SP 5	SP 6	SP 7	SP 8	SP 9
Side A	0.0110	0.0109	0.0108	0.0106	0.0110	0.0111	0.0107	0.0108	0.0109
Side B	0.0111	0.0109	0.0108	0.0108	0.0109	0.0109	0.0109	0.0108	0.0110

**Table 3:** Correction coefficients of each bending morphology.

Bending Shapes	Correction Coefficient
Type 1	*k*_1_ = 1.45
Type 2	*k*_2_ = 1.80
Type 3	*k*_3_^+^ = 1.49, *k*_3_^−^ = 1.10

**Table 4:** Mechanical property parameters of ABS materials.

Parameters	Value
elasticity modulus	2.2GPa
shearing modulus	318.9MPa
mass density	1020Kg/m^3^
tensile strength	30MPa
Poisson ratio	0.394

**Table 5:** Correction coefficients of each bending morphology.

Bending Shapes	Correction Coefficient
Type 1	*k*_1_ = 1.15
Type 2	*k*_2_ = 1.25
Type 3	*k*_3_^+^ = 1.15, *k*_3_^−^ = 1.17
Type 4	*k*_4_^+^ = 1.14, *k*_4_^−^ = 1.18
Type 5	*k*_5_^+^ = 1.19, *k*_5_^−^ = 1.21

**Table 6:** The standard deviation of the displacement at each measuring point (MP) under different bending shapes.

Bending Shapes	MP 1	MP 2	MP 3	MP 4	MP 5	MP 6	MP 7	MP 8	MP 9
Type 1	0.01	0.05	0.11	0.18	0.27	0.37	0.49	0.61	0.74
Type 2	0.02	0.08	0.15	0.22	0.30	0.37	0.43	0.50	0.56
Type 3	0.02	0.06	0.12	0.17	0.19	0.20	0.18	0.15	0.11

**Table 7:** The relative error percentages of the displacements at each measuring point (MP) after classification correction.

Bending Shapes	MP 1	MP 2	MP 3	MP 4	MP 5	MP 6	MP 7	MP 8	MP 9
Type 1	3.43	3.00	4.90	4.52	6.39	5.38	5.16	5.68	4.68
Type 2	2.02	1.94	3.92	5.27	3.21	2.03	3.16	4.67	7.04
Type 3	1.04	5.86	7.02	3.76	2.38	0.35	1.66	4.15	6.74
